# Validation of the nurse directed frailty assessment tool, to identify patients at risk of emergency department visits, hospitalisation, and 1-year all-cause mortality

**DOI:** 10.1007/s41999-025-01182-3

**Published:** 2025-03-14

**Authors:** P. H. Drop, C. van Ham, A. C. M. Mulder, P. A. Veeken – Dijkstra, J. O. Daal, L. A. R. Zwart

**Affiliations:** 1 Department of Geriatric Medicine, Dijklander Hospital, Hoorn, The Netherlands; 2 Department of Ageing and Public Health, Amsterdam University Medical Centres, Amsterdam, The Netherlands

**Keywords:** Frailty, Frailty assessment tool, Older patients, Frailty index

## Abstract

**Methods:**

This is a validation study of a new frailty assessment that can be administered by outpatient care nurses. The nurse directed frailty assessment (NDFA) encompasses the medical, psychological, social, and functional domain, based on standard care, and can be performed without any specialised equipment. Performance of the NDFA is compared to a comprehensive geriatric assessment (CGA)-based frailty index (FI), with generalised linear model with reporting of hazard ratios (HR) and 95% confidence intervals (95% CI). The best cutoff value for the NDFA was assessed by Youden index and the area under the receiver operator curve (ROC).

**Results:**

Within 1 year, 15 patients (5%) died, 57 (18%) had an unplanned hospital admission, and 83 (26%) visited the emergency department (ED). Based on the Youden index and ROC curve, the best cutoff value for the NDFA was 4 points. With a binary logistic regression model, an HR of 3.59 (95% CI 1.16–11.15, *p* < 0.001) was found for mortality. In the general mixed model with Poisson logistic regression, an HR of 1.78 (95% CI 1.06–2.97, *p* 0.028) was found for unplanned hospital and an HR of 1.87 (95% CI 1.25–2.78, *p* 0.002) was found for ED visits. The HR and 95% CI of the FI were similar for all three outcome measures.

**Conclusions:**

The NDFA identifies patients at risk for hospitalisation, emergency department visits, and mortality within 12 months equally well as the FI. Further research is necessary to determine the effectiveness of the NDFA in other settings than the geriatric medicine outpatient population.

**Supplementary Information:**

The online version contains supplementary material available at 10.1007/s41999-025-01182-3.

## Key summary points

**Aim:**

Development of a new frailty assessment that can be administered by outpatient care nurses, the nurse directed frailty assessment (NDFA).

**Findings:**

The NDFA identifies patients at risk for hospitalisation, emergency department visits, and mortality within 12 months equally well as the comprehensive geriatric assessment (CGA)-based frailty index.

**Message:**

The NDFA can help with selection of patients that might benefit from a referral to a Geriatrician, and future research is necessary to determine the effectiveness of the NDFA in other settings than the geriatric medicine outpatient population.

**Supplementary Information:**

The online version contains supplementary material available at 10.1007/s41999-025-01182-3.

## Introduction

With an ageing population, the prevalence of frailty will increase [1]. In 2050, 459 million people will be over 80 years old [2]. In the Netherlands, it is predicted that 25% of the population will be over 65 years old [3].

Frailty is defined as ‘a clinical state in which there is an increase in an individual’s vulnerability to developing adverse health-related events (including disability, hospitalizations, institutionalizations, and death) when exposed to endogenous or exogenous stressors’ [4]. Estimates around the prevalence of frailty and survival vary between studies, due to some part because definitions of frailty vary. Identifying frailty in individual patients can help in delivering patient-centred care. This might prevent harm and lead to better outcomes [1]. The comprehensive geriatric assessment (CGA) is considered the golden standard to assess frailty, and foremost to aid patient specific treatment decisions that addresses patient’s complaints and frailty [5, 6]. While a CGA is considered the golden standard by which to assess frailty, it is time consuming. To overcome this problem, various screening tools have been developed.

In 2001, the first assessment model was proposed by Fried and colleagues, which assessed weight loss, grip strength, self-reported exhaustion, walking time and activity [7, 8]. This was followed in the same year by model by Rockwood and colleagues who proposed a frailty index. In this frailty index, a cumulation of 80 deficits is analysed, ranging for depression to somatic illnesses. Later this was brought back to 40 deficits, including grip strength, pace, history of several illnesses and help in ADL tasks. This created frailty index shows a strong correlation with adverse outcomes [7, 9–11]. Since then, many screening and assessment tools were developed [7]. For an assessment of frailty, a tool preferably takes into account all four domains (physical, psychological, functional and social), and does not overly consume time or effort. A frailty index can be time consuming, but gives a more nuanced assessment than the Fried frailty phenotype. Fried’s phenotype is easily applied, but requires additional equipment, such a handgrip strength measurement [7, 12, 13]. The majority of screenings tools do not assess all four domains, commonly used in geriatric assessment [13, 14]. Especially factors within the psychosocial domain are often absent, even though they are known to be associated with functional frailty outcomes [14]. A CGA-based FI covers all domains and, when designed properly, can be used to compare frailty in between cohorts, even if the indexes themselves are not precisely similar [9, 10]. The true golden standard would be the CGA, and the FI comes closest. However, since a FI cannot be performed bedside, a need for easily and rapidly to perform screening tools remain.

For health care providers, it will become increasingly important to be able to identify which patients will benefit from demanding treatment regimes, such as major surgery and chemotherapy. Ideally, all older patients in need of such treatments would be offered a full comprehensive geriatric assessment, but this is not feasible at the moment, and will be only more challenging with the coming ageing of society [1]. With the nurse directed frailty assessment (NDFA), we aim to address a number of challenges concerning frailty assessment. It has been designed to be carried out by an outpatient nurse, of any department, without validation of a doctor. It makes use of the medical file and standard care, and does not require any additional tools or instruments, minimising the time investment needed. Instead of on the medical domain, the emphasis of the NDFA will lie in the psychological, functional and social domain. The NDFA will be validated against a CGA-based FI [9, 11]. The associations with mortality, unplanned hospital admissions, and emergency department visits will be analysed.

## Methods

### Patient cohort

To validate the NDFA, data of the participants of one centre of the Dutch-GERAF study are used, and the design of the study has been published [15]. The Dutch-GERAF study is a multicentre study into opportunistic screening for atrial fibrillation using a photoplethysmography (PPG) smartphone application that included 952 geriatric outpatients. All patients referred to the outpatient clinic were eligible for participation, and exclusion criteria were a severe dementia defined as a Mini Mental State Examination (MMSE), or Montreal Cognitive Assessment (MoCA) score of less than 15 points, a severe tremor, or not to be able to perform at least 3 PPG recordings. At baseline, all patients underwent a comprehensive geriatric assessment (CGA), with calculation of a frailty index (FI) based on the accumulation of deficits model [9, 11]. This FI consists of 46 factors, if absent factors are scored 0, if present factors are scored either 1 or 2, and add up to a maximum score of 51 points, and this specific FI has been applied in previous studies, and has shown a strong correlation with mortality [15–18]. The FI is calculated as the sum of factors present, their Frailty score, divided by 51, and patients are classified as robust if the FI is below 0.18, moderately frail with a FI from 0.18 to 0.25, and severely frail with a FI above 0.25. The FI has values between 0 and 1, resulting in a hazard ratio (HR) corresponding to an increase in FI from 0 to 1, which does not occur within the data and makes interpretation difficult. For this reason, the regression analysis is performed on the Frailty score (the sum of factors within the FI), this way the HR corresponds with 1 extra factor within the FI.

### The nurse directed frailty assessment

**Fig. 1:**
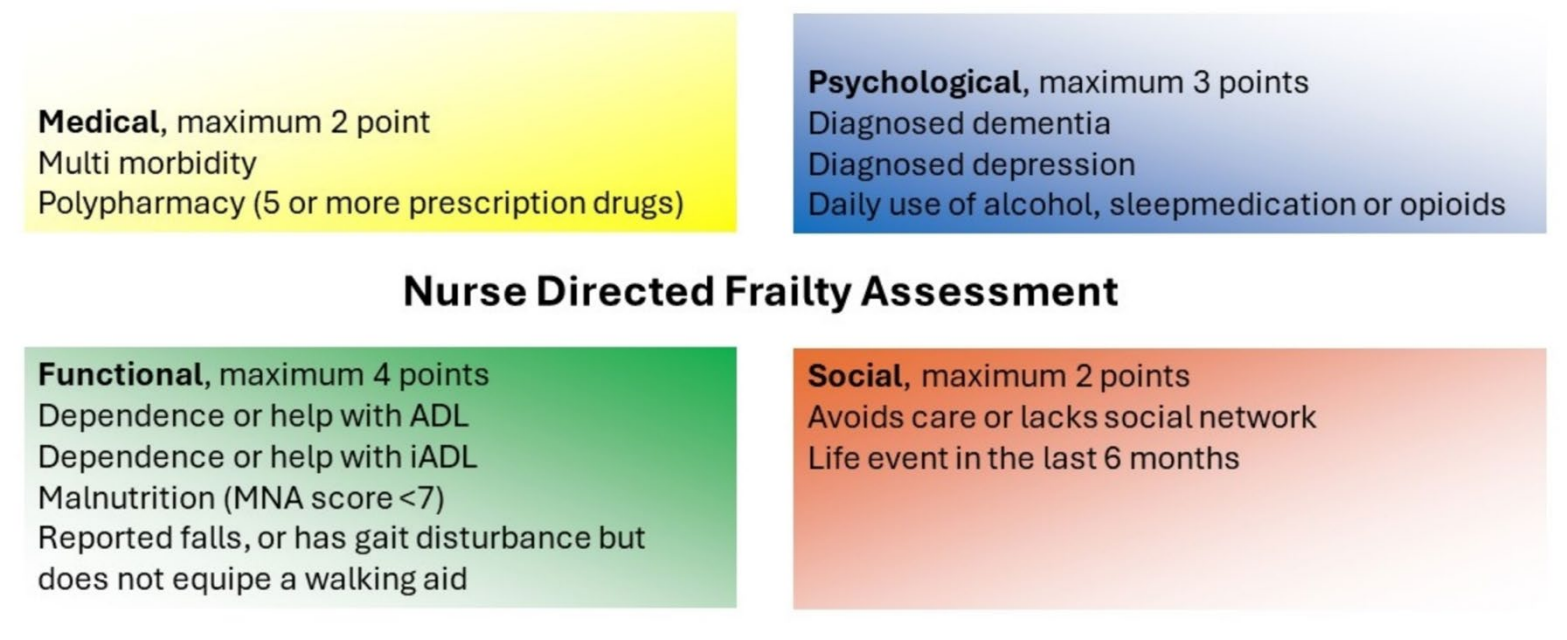
Domains and items in the nurse directed frailty assessment

This is the first validation study of the NDFA. The tool attempts to reflect the Geriatrics nurse clinical assessment, covering the four domains of the CGA. It is composed of information that is readily available and does not require a physicians’ confirmation or supervision. In its design, it enables any outpatient clinic nurse to perform the frailty assessment at the outpatient clinic independently, and without the use of specialised equipment. With its focus on readily available information, the extra effort of performing the assessment is minimised. By design, it does not include age or sex as criteria, acknowledging that younger people can also be frail. The NDFA encompasses the medical (2 points), psychological (3 points), social (2 points), and functional domain (4 points), as shown in Fig. 1. All items within each domain should be scored as present or absent, receiving 1 point if they are present.

Medical domain: multimorbidity, 1 point, defined as at least 2 chronic conditions. In addition, polypharmacy, 1 point, defined as the use of 5 different prescription drugs, including over the counter painkillers (excluding topically applied solutions) [19].

Psychological domain: known diagnosis of dementia, 1 point. The patient is currently in treatment for a depression, 1 point. In addition, the daily use of alcohol, opioids, or sleep medication, 1 point.

Functional domain: dependence on others for Activities of Daily Life (ADL), 1 point, defined as receiving any help in ADL by a spouse or caretaker. Dependence on others for Instrumental Activities of Daily Life (IADL), 1 point, defined as receiving any help with IADL task by a spouse or caretaker. The patient being at risk for malnourishment, 1 point, defined as having a Mini Nutritional Assessment score of 7 or less [ref 14]. In addition, the patient has a gait disturbance but does not make use of a walking aid, or reports falls despite a walking aid, 1 point.

Social domain: patients experienced a life event in the last 6 months (example given: loss of a child or partner, new diagnosis of threatening disease, divorce, financial setback), 1 point. In addition, they lack a social or formal network to rely on, or refuse professional help at home even though this would be beneficial, 1 point.

### Statistics and analysis

The primary outcomes are the hazard ratio’s (HR) and predictive capability of the NDFA for unplanned hospital admission, emergency department (ED) visits, and mortality at 6- and 12-month follow-up, as compared to the FI. Mortality will be reported as a rate, and both unplanned hospital admissions and ED visits will be reported as a count measure. All outcome measures have been collected by the principal GERAF investigators, directly from the individual patients’ electronical medical records, evaluating day-to-day notes and discharge letters.

For mortality, the HR is calculated with a multiple logistic regression analysis, adjusted for age and sex. For unplanned hospital admissions, or ED visits, the HR was calculated with a generalised linear model with Poisson regression, adjusted for age and sex. Either hospitalisation or ED visits could range from 0 to 2 events, registered as 0 for no hospitalisation/ED visit, 1 for a hospitalisation/ED visit in the first or second 6 months of the follow-up, or 2 in case there was a hospitalisation/ED visit in both periods of 6 months. It is possible that patients had multiple hospitalisations or ED visits in either 6-month periods; for this analysis, however, these would be registered as 1 event per 6-month period.

To compare the NDFA with the FI, The Akaike’s information criterion (AIC) was applied to evaluate and compare the goodness of fit between the NDFA and the FI, a difference of 2 or more units in the AIC will be considered significant. The area under the curve (AUC) of the receiver-operating characteristic curves (ROC) for the NDFA and FI to predict mortality will be calculated. To determine the sensitivity and specificity of the NDFA, the optimal cutoff score will be determined with the Youden index based on the ROC curve. For this cutoff value, the HR for mortality, unplanned hospital admissions, and ED visits will also be calculated. Afterwards, the sensitivity, specificity, positive and negative predictive value of the NDFA score at the cutoff value will be calculated. Statistical analyses will be performed in SPSS for Windows version 20.

## Results

The GERAF study included 952 patients, of which 314 were included at the Dijklander Hospital. Patients’ demographics and baseline characteristics are reported in Table 1. In summary, the median age was 78 ± 6.3 years, 171 (55%) were female, the median number of drugs was 6 ± 4.1 and polypharmacy was present in 180 (57%) patients. As expected for a geriatric cohort, the prevalence of cardiovascular morbidity was high. The five most prevalent morbidities were hypertension (58%), diabetes mellitus (27%), previous stroke (23%), atrial fibrillation (21%), and ischaemic heart disease (19%). Furthermore, 123 (39%) patients reported falls, and 149 (48%) had cognitive disorders (79 mild cognitive impairment, 26%, and 70 a dementia, 22%). The FI was normally distributed, with a mean of 0.17 ± 0.09. There were 191 (62%) robust patients at baseline, 59 (19%) had moderate frailty, and 61 (19%) of patients were severely frail. The NDFA had a normal distribution as well, the median score was 3 ± 1.4 points. The descriptive information of the NDFA and FI are shown in Supplementary Table 1.

**Table 1:** Baseline characteristics

	Total, *n* = 314
General features	
Age, median (mean, ± sd)	78 (78.7, 6.3)
Female sex, *n* (%)	171 (54.5)
Number of prescription drugs, median (mean, ± sd)	6 (6.9, 4.1)
Polypharmacy, *n* (%)	180 (57.3)
Systolic blood pressure, mean (± sd mmHg)	157 (24.6)
Diastolic blood pressure, mean (± sd mmHg)	82.8 (11.1)
Body mass index, kg/m^2^, mean (± sd)	26.7 (4.3)
Alcohol	
Not at all, *n* (%)	161 (51.3)
1–2 units per day, *n* (%)	109 (34.7)
More than 2 units per day, *n* (%)	28 (8.9)
Former alcohol abuse, *n* (%)	16 (5.1)
Smoking	
Never, *n* (%)	153 (48.7)
In the past, *n* (%)	137 (43.6)
Active smoker, *n* (%)	24 (7.6)
Atrial fibrillation, *n* (%)	65 (20.7)
Hypertension, *n* (%)	181 (57.6)
Hypercholesterolemia, *n* (%)	53 (16.9)
Diabetes mellitus, *n* (%)	84 (26.8)
Stroke in medical history, *n* (%)	73 (23.2)
Major bleeding in medical history, *n* (%)	30 (9.6)
Heart failure, *n* (%)	24 (7.6)
Ischaemic heart disease or angina pectoris, *n* (%)	61 (19.4)
Peripheral arterial disease, *n* (%)	31 (9.9)
Thyroid disease, *n* (%)	58 (18.5)
Chronic obstructive pulmonary disease, *n* (%)	38 (12.1)
Asthma, *n* (%)	10 (3.2)
Obstructive sleep apnoea syndrome, *n* (%)	19 (6.1)
Depression, *n* (%)	47 (15.0)
Laboratory	
Haemoglobin level, in mmol/L, mean (± sd)	8.5 (1.0)
Anaemia, sex adjusted, *n* (%)	100 (31.8)
Estimated glomerular filtration rate, in mL/min, mean (± sd)	68.5 (17.6)
Geriatric features	
Frailty index, mean (± sd)	0.17 (0.09)
Frailty categories	
Robust, *n* (%)	194 (61.8)
Moderate frailty, *n* (%)	59 (18.8)
Severe frailty, *n* (%)	61 (19.4)
Cognitive function	
Normal, *n* (%)	165 (52.5)
Mild cognitive impairment, *n* (%)	79 (25.5)
Dementia, *n* (%)	70 (22.3)
MMSE score, mean (± sd)	24.5 (3.6)
MoCA score, mean (± sd)	22.3 (4.2)
Diminished hand grip strength, physicians evaluation, *n* (%)	59 (18.8)
Gait disorder, *n* (%)	131 (41.7)
Walking aid, *n* (%)	101 (32.2)
Falls, *n* (%)	123 (39.2)
Parkinsonism, *n* (%)	30 (9.6)
Dependence in ADL, *n* (%)	55 (17.5)
Dependence in iADL, *n* (%)	115 (36.6)
Visual impairment, *n* (%)	54 (17.2)
Hearing impairment, *n* (%)	60 (19.1)

Within 1 year, 15 patients died (5%). A total of 57 patients (18%) experienced unplanned hospital admissions, of which 11 patients (4%) were admitted both within the first and second 6 months of follow-up. Eighty-three patients (26%) visited the emergency department within a year, and 25 of those (8%) both within the first and second 6 months of follow-up. These primary outcomes are summarised in Table 2.

**Table 2:** Mortality, hospital admissions, and emergency department visits

	6 months, *n* (%)	12 months, *n* (%)	Event at both 6 and 12 months, *n* (%)	Event at 6 or 12 months, *n* (%)
Mortality	5 (1.6)	10 (3.2)	n.a	15 (4.8)
Unplanned hospital admission	34 (10.8)	34 (10.8)	11 (3.5)	57 (18.1)
Emergency department visit	51 (16.2)	58 (18.5)	25 (8.0)	83 (26.4)

To analyse the association of the frailty score and NDFA score with unplanned hospital admissions or emergency department (ED) visits, a general linear mixed model with Poisson logistic regression was applied. In Table 3, the outcomes adjusted for age and sex are presented. An increase of one point in the frailty score was associated with a hazard ratio (HR) of 1.05 (95% CI 1.00–1.11, *p* = 0.07) for unplanned hospital admissions and 1.07 (95% CI 1.03–1.11, *p* = 0.001) for emergency department (ED) visits. Similarly, each additional point in the NDFA corresponded to an HR of 1.22 (95% CI 1.02–1.45, *p* = 0.03) for unplanned hospital admissions and 1.20 (95% CI 1.05–1.38, *p* = 0.01) for ED visits. For both outcomes, the NDFA demonstrated a better model fit than the frailty score, as shown by significantly lower Akaike information criterion (AIC) values: 346.6 vs. 361.9 for unplanned hospital admissions and 469.8 vs. 481.0 for ED visits. Regarding mortality, the frailty score was associated with an HR of 1.1 per additional point (95% CI 0.98–1.25, *p* = 0.09), while the NDFA yielded an HR of 1.4 per additional point (95% CI 0.94–2.14, *p* = 0.10).

**Table 3:** Association of the frailty score and NDFA with unplanned hospital admissions and emergency department visits

Primary outcome, adjusted for age and sex	Frailty score				NDFA			
	HR	95% CI	*p*	AIC	HR	95% CI	*p*	AIC
Unplanned hospital admissions	1.05	1.00–1.11	0.07	361.94	1.22	1.02–1.45	0.03	346.62
Emergency department visits	1.07	1.03–1.11	0.001	481.02	1.20	1.05–1.38	0.01	469.82

The ROC curves and area under the curves of the FI (A) and NDFA (B) for mortality within 12 months are shown in Supplementary Fig. 1. For the FI, the area under the ROC curve was 0.68, for the NDFA the area under the ROC curve was 0.66. Based on the Youden index and ROC curve for mortality, the best cutoff value for the NDFA was 4 points. After applying this cutoff value, in a binary logistic regression model, an HR of 3.59 (95% CI 1.16–11.15, *p* < 0.001) was found for mortality. In the general mixed model with Poisson logistic regression, an HR of 1.78 (95% CI 1.06–2.97, *p* 0.028) was found for unplanned hospital admissions, and an HR of 1.87 (95% CI 1.25–2.78, *p* 0.002) was found for ED visits, and is summarised in Table 4. At a cutoff value of 4 points, the sensitivity, specificity, negative and positive predictive values of the NDFA are presented in Table 5. On all three outcomes, the specificity and negative predictive values were good or high, while the sensitivity and positive predictive values were low.

**Table 4:** Performance of NDFA at a cutoff value of 4 points

Binary logistic regression, adjusted for age and sex	HR	95% CI	*p*
All-cause mortality, within 12 months	3.59	1.16–11.15	< 0.001
General mixed model, Poisson logistic regression, adjusted for age and sex			
Unplanned hospital admissions, as a count	1.78	1.06–2.97	0.028
Emergence department visits, as a count	1.87	1.25–2.78	0.002

**Table 5:** Sensitivity, specificity, negative and positive predictive values of the NDFA at a 4-point cutoff

	Sensitivity	Specificity	Positive predictive value	Negative predictive value
Mortality	0.60	0.74	0.11	0.97
Unplanned hospital admissions	0.33	0.74	0.21	0.84
Emergency department visits	0.40	0.77	0.38	0.78

## Discussion

In this study, we introduce a short and easily applicable frailty assessment, which can be performed by any outpatient care nurse. The NDFA was validated against a CGA-based FI, and the NDFA has shown similar to better predictive capabilities, for the key outcomes unplanned hospital admission, and emergency department visits, at both 6 and 12 months. Furthermore, at a cutoff value of 4 points, a HR for mortality adjusted for age and sex was found of 3.6 (95% CI 1.2–11.2, *p* < 0.001), illustrating that the NDFA indeed identifies frail patients.

The NDFA simplifies the assessment of frailty using readily available information, thus saving time and effort while still covering key domains of the geriatric assessment. Moreover, the NDFA is designed to be intuitive for nurses with minimal additional training and does not require special equipment, reducing reliance on self-report and potentially increasing accuracy through professional observation. Applying the NDFA at outpatient clinics can help treating physicians to select which patients might benefit from a full CGA and referral to a geriatrician.

The association with mortality is helpful in determining if frailty assessments in general perform as expected [7, 9, 10, 12]. The similar AUC of the ROC curves for the 1-year all-cause mortality suggests a moderate performance of both assessments to differentiate between patients at increased risk, and those without. However, for very old and frail patients, mortality might not be an avoidable outcome, and outcomes as ED visits and hospital admission become more relevant. When comparing the AIC values and HRs, here the NDFA seems to outperform the applied FI. As compared to the Tilburg Frailty Indicator (TFI) and the Edmonton Frail Scale (EFS), the NDFA demonstrated reasonable sensitivity and specificity for predicting outcomes [20–22]. Importantly, the negative predictive value of the NDFA was good for emergency department visits and hospitalisation, and excellent for mortality. This holds promise to apply the NDFA as a tool to rule out frailty, and aid in the selection of patients for whom a CGA will have the most clinical benefits. Especially the application of the NDFA at non-geriatric medicine outpatient clinic holds the potential to identify patients at risk to be unknowingly frail. Were those patients to be referred for a full CGA, potential medication adjustments might be identified, but also cognitive analysis if necessary, malnutrition addressed, sarcopenia recognised and improved with physiotherapy [5]. Future research should go beyond only the identification of frailty, and focus on the effects on relevant outcomes such as hospital admission, and ED visits, after frailty has been recognised and a CGA-based patient-centred treatment plan has been made.

Strengths of the study are the comparison of the NDFA with a CGA-based FI, providing a clear benchmark for evaluating the performance and validity of the NDFA. The NDFA itself has been prospectively designed, which increases the likelihood of external validity in cohorts other than the geriatric outpatient population. Nevertheless, the current findings are limited to the geriatric outpatient population and should not be generalised to other patient population without external validation. All patients in this cohort received a full CGA, including a mediation review and personalised treatment plan. Correlations of the NDFA could either be stronger or weaker in other cohorts, as non-geriatric outpatient cohorts are less likely to be frail (selection bias). On the other hand, the opposite, if frailty is not recognised in other outpatient clinic cohorts, the possible benefit of a CGA might even be bigger. Whether the predictive qualities of the NDFA are also valid in other outpatient populations needs to be confirmed by future studies. Another limitation within this study was that admissions and hospitalisations within the first and second 6 months of follow-up were registered as that they occurred, or not. The occurrence of multiple visits or admissions was not clearly registered in these data, leading to a maximum count of visits/admission of 2, also if truly it was, for example, 5. Thereby, the association with the FI and NDFA with emergency department visits and unplanned hospital admissions were possibly underestimated. Last, as with all tools, while the NDFA includes multiple frailty domains, some relevant factors may not have been captured, potentially limiting its predictive accuracy in diverse clinical populations.

## Conclusion

The NDFA successfully identifies patients at risk for hospitalisation, emergency department visits, and mortality within 12 months. With a good negative predictive value at a cutoff value of 4 points, ranging from 78% for ED visits, and 97% for mortality, it can be of aid health to care professionals in selecting patients that could benefit from a full CGA and referral to a geriatric medicine service. Its design makes it easily implemented by all outpatient care nurses, as it is based on usual care, without the need for specialised equipment. This overcomes barriers such as additional training, or the time investment of a full geriatric workup. Further research is necessary to determine the effectiveness of the NDFA in other settings than the geriatric medicine outpatient population.

## Supplementary Information

Below is the link to the electronic supplementary material.

Supplementary file1 (DOCX 115 KB)

## Data Availability

Upon reasonable request and after approval of the GERAF researchers, it is possible to share a selection of anonymised data.
