# Supplementary material for: Validation of the nurse directed frailty assessment tool, to identify patients at risk of emergency department visits, hospitalisation, and 1-year all-cause mortality
Source: Eur Geriatr Med. 2025 Mar 14;16(3):1037–44. doi: 10.1007/s41999-025-01182-3 (PMC12174208; doi:10.1007/s41999-025-01182-3)
Supplement: Supplementary file 1 — Supplementary file1 (DOCX 115 KB) [file 41999_2025_1182_MOESM1_ESM.docx]

**Supplementary Materials**

**Supplementary Table 1. NDFA and FI score**

| Domain | mean | median | sd |
| --- | --- | --- | --- |
| Medical | 1.42 | 2 | 0.69 |
| Psychological | 0.5 | 0 | 0.62 |
| Functional | 0.19 | 1 | 0.84 |
| Social | 0.74 | 0 | 0.40 |
| Overall NDFA score | 2.66 | 3 | 1.42 |
|  |  |  |  |
| Frailty index | 0.17 | 0.16 | 0.09 |

**Supplementary Figure 1.**


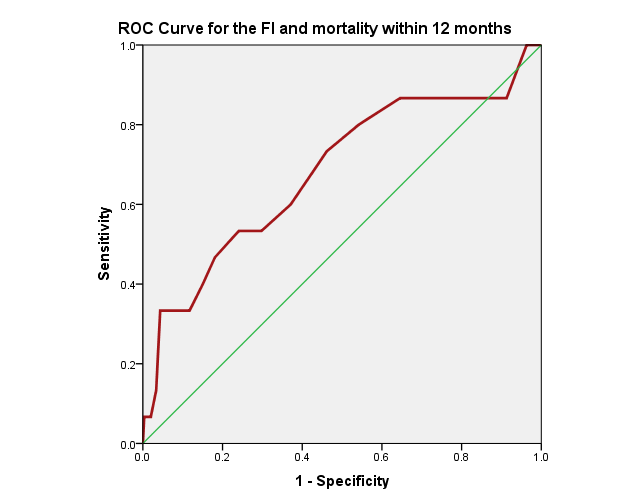


**Supplementary Figure 1. The ROC curves of the Frailty Index and NDFA**

**A**

A: FI and mortality within 12 months. B: NDFA : and mortality within 12 months.

Area under the curve: 0.68

Area under the curve: 0.66


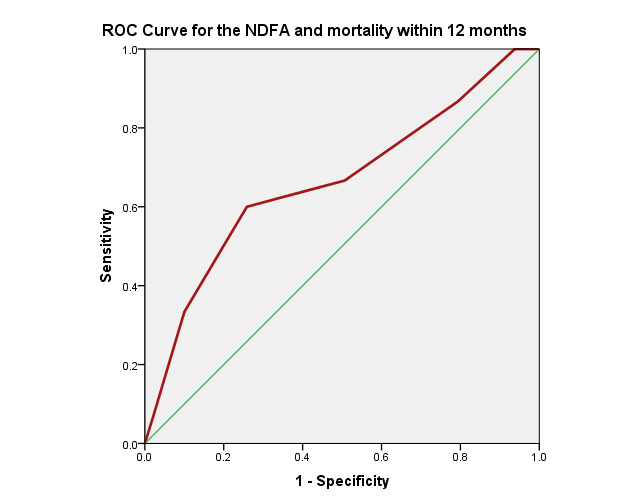


**B**
